# Association of the *IL2RA/CD25* Gene With Juvenile Idiopathic Arthritis

**DOI:** 10.1002/art.24187

**Published:** 2009-01

**Authors:** Anne Hinks, Xiayi Ke, Anne Barton, Steve Eyre, John Bowes, Jane Worthington, Susan D Thompson, Carl D Langefeld, David N Glass, Wendy Thomson, the UK Rheumatoid Arthritis Genetics Consortium, the British Society of Paediatric and Adolescent Rheumatology Study Group

**Affiliations:** 1 University of ManchesterManchester, UK; 2 Cincinnati Children's Hospital Medical CenterCincinnati, Ohio; 3 Wake Forest University School of MedicineWinston-Salem, North Carolina

## Abstract

**Objective:**

*IL2RA/CD25*, the gene for interleukin-2 receptor α, is emerging as a general susceptibility gene for autoimmune diseases because of its role in the development and function of regulatory T cells and the association of single-nucleotide polymorphisms (SNPs) within this gene with type 1 diabetes mellitus (DM), Graves' disease, rheumatoid arthritis (RA), and multiple sclerosis (MS). The aim of this study was to determine whether SNPs within the *IL2RA/CD25* gene are associated with juvenile idiopathic arthritis (JIA).

**Methods:**

Three SNPs within the *IL2RA/CD25* gene, that previously showed evidence of an association with either RA, MS, or type 1 DM, were selected for genotyping in UK JIA cases (n = 654) and controls (n = 3,849). Data for 1 SNP (rs2104286) were also available from North American JIA cases (n = 747) and controls (n = 1,161). Association analyses were performed using Plink software. Odds ratios (ORs) and 95% confidence intervals (95% CIs) were calculated.

**Results:**

SNP rs2104286 within the *IL2RA/CD25* gene was significantly associated with UK JIA cases (OR for the allele 0.76 [95% CI 0.66–0.88], *P* for trend = 0.0002). A second SNP (rs41295061) also showed modest evidence for association with JIA (OR 0.80 [95% CI 0.63–1.0], *P* = 0.05). Association with rs2104286 was convincingly replicated in the North American JIA cohort (OR 0.84 [95% CI 0.65–0.99], *P* for trend = 0.05). Meta-analysis of the 2 cohorts yielded highly significant evidence of association with JIA (OR 0.76 [95% CI 0.62–0.88], *P* = 4.9 × 10^−5^).

**Conclusion:**

These results provide strong evidence that the *IL2RA/CD25* gene represents a JIA susceptibility locus. Further investigation of the gene using both genetic and functional approaches is now required.

Juvenile idiopathic arthritis (JIA), the most common chronic rheumatic disease in children (1), is an umbrella term for diseases that start before the age of 16 years and are characterized by arthritis that persists for more than 6 weeks. JIA can be subdivided into 7 clinically more homogeneous subtypes, using the International League of Associations for Rheumatology (ILAR) classification system (2).

The genetic basis of JIA is complex, but it has been estimated that the sibling recurrence risk (λ_s_) is 15 (3). The most well-established genetic factor for JIA is *HLA*, and associations with *HLA* class I and class II genes exist, although both the strength of the associations and the associated alleles vary between subtypes (4). Recently, a variant in the coding region of the *PTPN22* gene, which has been found to be associated with a number of autoimmune diseases, including rheumatoid arthritis (RA), type 1 diabetes mellitus (DM), autoimmune thyroid disease (AITD), and systemic lupus erythematosus (SLE) (5), was identified as a second susceptibility locus for JIA (6). The effect size for *PTPN22* varies between subtypes but, in general, is smaller than that for *HLA*, and it has been hypothesized that there are additional genetic risk factors for JIA that remain to be discovered.

Another gene that is emerging as a potential “autoimmune gene” is *IL2RA/CD25*. It was initially identified as a candidate gene for type 1 DM and AITD (7). Further support has come from recently conducted genome-wide association studies of RA (8) and multiple sclerosis (MS) (9) showing that the same SNP, rs2104286, which lies within intron 1 of the *IL2RA/CD25* gene (8), is associated with both diseases. The study in MS patients also identified an association of an additional SNP, rs12722489, in linkage disequilibrium with it (9). More recently, the *IL2RA/CD25* region has been intensely studied in type 1 DM, where large-scale fine-mapping across the *IL2RA/CD25* gene found strong statistical evidence of association with 2 independent groups of SNPs (10). In that study, the presence of the susceptibility alleles was also shown to be correlated with lower concentrations of soluble interleukin-2 receptor α (IL-2Rα)/CD25.

The *IL2RA/CD25* gene encodes 1 of the subunits of the IL-2 receptor that binds IL-2 and is vital in the regulation of T cell function. IL2RA/CD25–knockout mice develop severe systemic autoimmune disease, a paradoxical finding suggesting that the gene is needed for down-regulation of immune responses in order to prevent autoimmunity. IL-2Rα/CD25 is the hallmark antigen of regulatory T cells (11–14), which play a vital role in the suppression of autoreactive T cells that escape other methods of tolerance. Depletion of these cells in mouse models results in the spontaneous development of autoimmune diseases (11). Thus, the evidence is building in support of a critical role of the IL-2/IL-2Rα–dependent regulatory pathway in the development of autoimmunity.

Given the evidence of a role of variation in the *IL2RA/CD25* gene in autoimmune disease susceptibility provided by the genome-wide association studies of MS and RA and the fine-mapping of the *IL2RA/CD25* region in type 1 DM, we hypothesized that *IL2RA/CD25* may also play a role in JIA. The aim of this study, therefore, was to determine whether *IL2RA/CD25* SNPs previously found to be associated with RA, MS, and type 1 DM are also associated with JIA.

## PATIENTS AND METHODS

### UK patients and controls

DNA was available for 654 white UK JIA patients from the British Society for Paediatric and Adolescent Rheumatology (BSPAR) National Repository for JIA. JIA cases were classified according to ILAR criteria (15). This is a combined set of all ILAR subtypes, including systemic-onset JIA (n = 115), persistent (n = 194) and extended oligoarthritis (n = 86), rheumatoid factor (RF)–negative (n = 138) and RF-positive (n = 35) polyarthritis, enthesitis-related JIA (n = 28), psoriatic JIA (n = 51), and cases with peripheral synovitis, which could not be classified (n = 7).

DNA samples from 3,849 healthy white control subjects were available from 5 centers in the UK (16), as follows: Manchester (n = 924), Sheffield (n = 995), Leeds (n = 532), Aberdeen (n = 862), and Oxford (n = 536). All study subjects were recruited with approval of the local ethics committees (North-West Multi-Centre Research Ethics Committee [MREC 99/8/84] and the University of Manchester Committee on the Ethics of Research on Human Beings) and provided informed consent.

### Genotyping of the UK cohort

Three *IL2RA/CD25* SNPs were selected for investigation: rs2104286, rs41295061, and rs11594656. SNP rs2104286 is the marker at the *IL2RA/CD25* locus that was most strongly associated with RA in the Wellcome Trust Case Control Consortium (WTCCC) genome-wide association study (8) and has also been associated with MS (9). SNPs rs41295061 and rs11594656 are the markers that were most significantly associated in each of the 2 independent groups of SNPs identified in the type 1 DM fine-mapping study of *IL2RA/CD25* (10). Genotyping of the UK JIA cases and controls was performed using the Sequenom iPLEX platform (Sequenom, San Diego, CA). A 90% sample quality control rate and a 95% SNP genotyping success rate was imposed on the analysis.

Genotype data for SNP rs2104286 in the controls were combined with the data for that SNP obtained in the WTCCC genome-wide association study (8). The total sample size in the controls was 6,787.

### North American patients and controls

A second cohort comprising 747 white North American JIA patients and 1,161 white North American controls was also studied. The majority of the cases were recruited at the Cincinnati Children's Hospital Medical Center. The medical and clinical data for these study subjects are available in the Pediatric Rheumatology Research Registry maintained within the William S. Rowe Division of Rheumatology. The remaining subjects were recruited from collaborating centers across the US. These collections include the National Institute of Arthritis and Musculoskeletal and Skin Diseases–supported JIA Affected Sibpair Registry, a national collection of multiplex families that includes sample collections and clinical information. All patients met the ILAR criteria for JIA, which were assigned retrospectively; therefore, for the purposes of this study, a patient was considered to be RF-negative based on a single test. ILAR JIA subtypes in this group of patients consisted of persistent oligoarthritis (n = 325), extended oligoarthritis (n = 105), RF-positive polyarthritis (n = 4), RF-negative polyarthritis (n = 312), and systemic-onset JIA (n = 1).

As part of a genome-wide association study of JIA, the North American JIA cases had previously been genotyped for 1 of the *IL2RA/CD25* SNPs, rs2104286, using Affymetrix GeneChip Human Mapping 500K arrays (Affymetrix, Santa Clara, CA). This study was approved by the Institutional Review Board of Cincinnati Children's Hospital Medical Center. Appropriate consent was obtained from the subjects and their family members or an Institutional Review Board exemption was granted for legacy samples which predated the current consent process and which are now de-identified.

The controls comprised 1,161 healthy white individuals from the New York Cancer Project. The rs2104286 SNP had not been genotyped directly, but the imputed genotypes were inferred from multimarker combinations. Only samples with a >95% confidence score for their imputed genotypes were included (17).

### Statistical analysis

Genotype and allele frequencies were compared between JIA cases and controls using Stata version 9 SE (StataCorp, College Station, TX) and Plink (18) software, and odds ratios (ORs) and 95% confidence intervals (95% CIs) were calculated. Stratification by ILAR subtype, antinuclear antibody (ANA) status, and sex was performed. Conditional logistic regression was performed using Plink software. Haplotype analysis was performed using Haploview version 4.0 software (19). In addition, the Cochran-Mantel-Haenszel test was used to perform a meta-analysis of the 2 cohorts, and a test for heterogeneity between cohorts was performed using the Breslow-Day test.

## RESULTS

All SNPs were in Hardy-Weinberg equilibrium in the UK and North American control cohorts. In the UK cohort, SNP rs2104286, which lies within the *IL2RA/CD25* gene, was associated with JIA (OR for the allele 0.76 [95% CI 0.66–0.88], *P* for trend = 0.0002) (Table 1).

**Table 1:** Association of *IL2RA/CD25* SNPs in UK JIA cases and controls, and of SNP rs2104286 in North American JIA cases and controls*

							Genotype frequency, no. (%)							
									
Cohort, marker	Chr.	Minor/major allele	Genotyping success rate (%)	HWE in controls	MAF		Cases			Controls			*P* for trend	OR (95% CI) for allele
									
					Cases	Controls	11	12	22	11	12	22		
UK														
rs2104286	10	G/A	96.4	0.88	0.22	0.27	30 (5.1)	207 (34.9)	356 (60)	502 (7.6)	2,621 (39.9)	3,454 (52.5)	0.0002	0.76 (0.66–0.88)
rs41295061	10	A/C	94	0.61	0.07	0.09	2 (0.3)	87 (14.1)	530 (85.6)	32 (0.9)	591 (16.3)	2,991 (82.8)	0.05	0.8 (0.63–1.0)
rs11594656	10	A/T	94	0.56	0.24	0.25	35 (5.7)	223 (36.0)	361 (58.3)	212 (5.9)	1,356 (37.5)	2,046 (56.6)	0.73	0.95 (0.82–1.09)
North American														
rs2104286	10	G/A	96.6	0.88	0.23	0.26	34 (4.7)	262 (36.3)	426 (59.0)	70 (6.0)	454 (39.1)	637 (54.9)	0.05	0.84 (0.65–0.99)

* In the UK cohort, there were 654 juvenile idiopathic arthritis (JIA) cases and 3,849 controls. In the North American cohort, there were 747 JIA cases and 1,161 controls. SNPs = single-nucleotide polymorphisms; Chr. = chromosome; HWE = Hardy-Weinberg equilibrium; MAF = minor allele frequency; OR = odds ratio; 95% CI = 95% confidence interval.

JIA is a phenotypically heterogeneous disease, with a female predominance and an association with the presence of ANAs. Stratification analysis was therefore performed by ILAR subtype, sex, and the presence or absence of ANAs. Analysis stratifying by ILAR subtype (Table 2) showed that the association with SNP rs2104286 was strongest in both the persistent (OR for the allele 0.69 [95% CI 0.53–0.89], *P* for trend = 0.005) and extended (OR for the allele 0.53 [95% CI 0.35–0.81], *P* for trend = 0.003) oligoarthritis subtypes. The association of rs2104286 with JIA was also stronger in the ANA-positive subgroup (OR for the allele 0.65 [95% CI 0.49–0.86], *P* for trend = 0.002) and the female cases (OR for the allele 0.76 [95% CI 0.64–0.91], *P* for trend = 0.002) as compared with the controls (Table 2).

**Table 2:** Association analysis of *IL2RA/CD25* SNPs in UK and North American JIA cases stratified by ILAR-classified subtype, and in UK JIA cases and controls stratified by ANA status and sex*

			rs2104286		rs41295061		rs11594656	
					
	No. of cases	No. of controls	*P* for trend	OR (95% CI) for allele	*P* for trend	OR (95% CI) for allele	*P* for trend	OR (95% CI) for allele
UK cohort								
JIA subtype, by ILAR criteria								
Systemic	115	–	0.11	0.77 (0.56–1.1)	0.16	0.67 (0.39–1.17)	0.92	0.98 (0.72–1.34)
Persistent oligoarthritis	194	–	0.005	0.69 (0.53–0.89)	0.15	0.73 (0.49–1.11)	0.82	1.03 (0.81–1.31)
Extended oligoarthritis	86	–	0.003	0.53 (0.35–0.81)	0.11	0.57 (0.29–1.13)	0.61	0.91 (0.63–1.31)
RF-negative polyarthritis	138	–	0.57	0.92 (0.68–1.23)	0.52	1.14 (0.76–1.73)	0.43	0.88 (0.66–1.19)
Psoriatic	51	–	0.43	0.83 (0.52–1.32)	0.47	0.75 (0.35–1.63)	0.75	1.07 (0.69–1.69)
ANA status								
Positive	177	6,787	0.002	0.65 (0.49–0.86)	0.16	0.73 (0.48–1.13)	0.3	0.86 (0.64–1.15)
Negative	358	6,787	0.06	0.84 (0.7–1.0)	0.56	1.08 (0.84–1.38)	0.49	0.94 (0.78–1.13)
Sex								
Female	466	3,902	0.002	0.76 (0.64–0.91)	0.47	0.91 (0.69–1.18)	0.04	0.83 (0.69–0.99)
Male	207	2,675	0.05	0.78 (0.61–1.0)	0.02	0.59 (0.37–0.92)	0.06	1.25 (0.98–1.59)
North American cohort								
JIA subtype, by ILAR criteria								
Persistent oligoarthritis	325	–	0.09	0.83 (0.59–1.03)	–	–	–	–
Extended oligoarthritis	105	–	0.85	0.97 (0.57–1.26)	–	–	–	–
RF-negative polyarthritis	312	–	0.21	0.86 (0.6–1.07)	–	–	–	–

* SNPs = single-nucleotide polymorphisms; JIA = juvenile idiopathic arthritis; ILAR = International League of Associations for Rheumatology; ANA = antinuclear antibody; OR = odds ratio; 95% CI = 95% confidence interval; RF = rheumatoid factor.

Association analysis of the 2 type 1 DM–associated *IL2RA/CD25* SNPs revealed that only 1 of them, rs41295061, showed borderline evidence of association with JIA as compared with controls (OR for the allele 0.8 [95% CI 0.63–1.0], *P* for trend = 0.05) (Table 1). Analysis stratifying JIA cases by ILAR subtype (Table 2) showed no statistically significant differences between the subtypes. Logistic regression analysis conditioning on the most-associated SNP, rs2104286, suggested that this SNP accounts for the association with JIA and that the association with rs41295061 is not an independent association but is due to linkage disequilibrium with rs2104286 (*P* = 0.62 for rs41295061 after conditioning on rs2104286), despite only moderate correlation (r^2^ = 0.27). Haplotype analysis for the 2 SNPs showed that the haplotype of the 2 major alleles of both SNPs was overrepresented in cases compared with controls (OR 0.77 [95% CI 0.58–0.93], *P* = 0.0035), with an odds ratio similar to that for the single-marker association (OR 0.77 [95% CI 0.66–0.88]).

Genotype frequencies in the North American JIA cases were similar to those found in the UK cohort. Comparison of genotype frequencies between North American cases and controls provided further evidence of association of rs2104286 with JIA (OR for the allele 0.84 [95% CI 0.65–0.99], *P* for trend = 0.05) (Table 1). Stratification of the North American JIA cases by ILAR subtype was performed, and the association was strongest in the persistent oligoarthritis subtype (OR for the allele 0.83 [95% CI 0.59–1.03], *P* for trend = 0.09) (Table 2).

There was no significant evidence of heterogeneity between the UK and North American data sets. Meta-analysis of the 2 cohorts yielded highly significant evidence of association (OR 0.76 [95% CI 0.62–0.88], *P* = 4.9 × 10^−5^).

## DISCUSSION

In this study, we found significant evidence of association of a polymorphism within the *IL2RA/CD25* gene with JIA in 2 independent cohorts. SNPs within the *IL2RA/CD25* gene have previously been associated with a number of other autoimmune diseases, including type 1 DM (10), Graves' disease (20), and MS (9). In addition, a rare mutation of the *IL2RA/CD25* gene can lead to the development of a severe autoimmune disease characterized by decreased numbers of peripheral T cells displaying abnormal proliferation, with extensive lymphocytic infiltration of tissues accompanied by tissue atrophy and inflammation, but normal B cell development (21). The finding of an association with JIA further extends the evidence that variation across this gene predisposes to autoimmunity in general.

The concept of “autoimmune genes” is not novel. Previous analysis of linkage studies in a number of autoimmune diseases showed evidence of overlap between regions of linkage (22). Furthermore, there is precedent for this because the *PTPN22* gene has been associated with a variety of autoimmune diseases, although, interestingly and unlike the *IL2RA/CD25* gene, not with MS.

Three SNPs were selected for genotyping based on their association with other autoimmune diseases, and 2 of these SNPs were associated with JIA in the current study. The strongest effect was seen with rs2104286 (OR for minor allele 0.76 [95% CI 0.66–0.88]), which is comparable to the ORs calculated for MS (OR 0.81 [95% CI 0.74–0.89]) and RA (OR 0.81 [95% CI 0.73–0.89]). This SNP maps to intron 1 of the *IL2RA/CD25* gene, and was selected for genotyping because recent genome-wide association studies of both RA and MS found an association with the same variant (rs2104286). In addition, the MS study also found an association with a SNP in strong linkage disequilibrium with it, rs12722489 (9).

The other 2 SNPs genotyped in this study of JIA were selected based on recent studies of the *IL2RA/CD25* region in type 1 DM (10). Fine-mapping studies detected an association with 2 independent groups of SNPs spanning overlapping regions of 14 kb and 40 kb, encompassing *IL2RA/CD25* intron 1 and the 5′-intergenic region between *IL2RA/CD25* and *RBM17*. The first region contains a group of 8 genetically indistinguishable SNPs, the most strongly associated being rs41295061, while the second group comprised 3 indistinguishable SNPs, the most strongly associated being rs11594656. Both of these SNPs are potentially functional, given that genotypes at each have been correlated with lower concentrations of soluble IL-2Rα/CD25. This suggests that a reduction in immune activation may predispose to type 1 DM (10) and, indeed, other autoimmune diseases.

Interestingly, in the present study, only 1 of the 2 SNPs associated with type 1 DM (rs41295061) showed a weak association with JIA. The evidence of association with this SNP was stronger when genotype frequencies from the JIA cases were compared with those in the large type 1 DM control cohort (n = 6,855) (OR for the allele 0.69 [95% CI 0.55–0.85], *P* for the genotype = 0.002), while that of the second SNP (rs11594656) remained nonsignificant (OR for the allele 0.94 [95% CI 0.82–1.08], *P* for the genotype = 0.4). Association of the type 1 DM intron 1 SNP (rs41295061) with JIA was not as strong as with SNP rs2104286, and this association requires validation in an additional cohort. Hence, it will be vital to determine whether the genotype at the rs2104286 locus also correlates with soluble IL-2Rα/CD25 levels or any other functional effect.

Examination of linkage disequilibrium between the SNPs tested in this study suggested that there is low linkage disequilibrium between rs2104286 and the 2 SNPs (rs41295061 and rs11594656) identified in the type 1 DM fine-mapping study (r^2^ = 0.27 and r^2^ = 0.03, respectively). However, logistic regression analysis of the 3 SNPs conditioning on the most-associated SNP, rs2104286, suggests that this is the SNP that drives the association with JIA. The association with rs41295061 is due to linkage disequilibrium with rs2104286. Further investigation of SNPs across the *IL2RA/CD25* gene will now be required to identify the causal variant.

The association with rs2104286 was confirmed in an independent cohort of North American JIA cases and controls (OR 0.84 [95% CI 0.65–0.99], *P* for trend = 0.05). Meta-analysis of the 2 data sets strengthened the effect (OR 0.76 [95% CI 0.62–0.88], *P* = 4.9 × 10^−5^), thereby providing further support for the role of *IL2RA/CD25* in susceptibility to JIA.

False-positive associations can arise as a result of population stratification. The WTCCC study (8) previously established that there was very little evidence of this across the UK. We did not test the North American data for population stratification. However, there was no evidence of heterogeneity between the 2 populations as assessed by the Breslow-Day test. Furthermore, the fact that the genotype/allele frequencies for the associated markers were very similar in the UK and the North American case and control populations suggests that the detected association is genuine.

JIA is a phenotypically heterogeneous disease. Thus, although it may be argued that stratification analysis may result in inflated Type I error rates because of multiple testing, the creation of more homogenous subgroups may increase the power to detect an association. In the current study, evidence of an association with rs2104286 was strongest in the oligoarthritis subtypes, female cases, and JIA cases with ANAs. The association with all these subgroups is not surprising, since the phenotypes are correlated. The same subgroup effect does appear to be true for rs41295061. However, it should be noted that the small sample sizes in some of the ILAR subtypes may have limited the ability to draw robust conclusions about association of the *IL2RA/CD25* SNPs with those phenotypes. Comparison of rs2104286 SNP allele frequencies between the 5 subtypes suggests that they are not statistically different (*P* = 0.28). Therefore, larger sample sizes will be required to be able to clarify association across the individual subtypes.

The *IL2RA/CD25* gene was a strong candidate JIA susceptibility gene not only because of the previous evidence of association with a variety of other autoimmune diseases, but also because of its role in the T cell signaling pathway, which is thought to play a key role in mediating the inflammatory autoimmune response in JIA. IL-2, the T cell growth factor, exerts its effect on T cells through binding with the IL-2R. The IL-2 receptor is composed of 3 chains, α, β, and γ, the α-chain being encoded by the *IL2RA/CD25* gene. The α and β chains are involved in the binding of IL-2, and the β and γ chains are involved in signal transduction once IL-2 has bound. IL-2Rα/CD25 is central to immune regulation via its role in the development and function of regulatory T cells. These cells are increasingly seen as playing a central role in self tolerance. CD4+CD25+ Treg cells suppress activated T cells, and this immune suppression is vital in preventing autoimmune diseases. Hence, variation in the gene encoding IL-2Rα/CD25 could have profound effects in the predisposition to autoimmune disease.

A recent study investigated CD4+CD25+ Treg cells in children with persistent oligoarthritis (1–4 joints affected at diagnosis and throughout the disease course) and extended oligoarthritis (a more destructive disease course in which the number of affected joints increases beyond 4 within 6 months of disease onset), hypothesizing that Treg cells may play a role in the reversal of the autoimmune disease process in patients with persistent oligoarticular JIA (23). It was shown that the number of CD4^+^CD25^bright^ Treg cells at the site of inflammation (i.e., the joints) was enriched compared with the number in the peripheral blood. In addition, there was a greater frequency of CD4^+^CD25^bright^ Treg cells in patients with persistent oligoarthritis as compared with those with extended oligoarthritis. CD4^+^CD25^bright^ Treg cells in the synovial fluid (SF) showed higher expression of many activation markers, such as FoxP3, CTLA-4, and HLA–DR, suggesting that they are more functionally mature than those in the peripheral blood. These cells also showed an increased suppressive capacity in vitro compared with cells from the peripheral blood (23). A more recent study also found that the ratio of Treg cells to activated T cells was higher in patients with oligoarthritis than in patients with polyarthritis (24). Similar findings in RA have been reported, with higher numbers of Treg cells and concomitantly increased suppressive activity found in SF compared with peripheral blood cells (25). Thus, there appear to be functional differences in Treg cells across JIA subtypes.

Interestingly, when we analyzed the *IL2RA/CD25* SNPs by JIA subtype, the strongest association was in both oligoarthritis subtypes, although the limited sample sizes of some of the other subtypes make conclusions about subtype-specific associations difficult. In future studies, we will examine correlations between Treg cell function and *IL2RA/CD25* genotype.

The IL-2 receptor has already been a potential target of immunologic therapy in autoimmune diseases. For example, IL-2–directed immunotherapies have been successful in ameliorating disease in experimental autoimmune encephalomyelitis, an animal model of MS (26). In human MS, a monoclonal antibody that binds to the α-chain of IL-2R has been shown to have some therapeutic benefits, although its mechanism of action is unknown (27).

In summary, we identified a putative novel JIA susceptibility gene, *IL2RA/CD25*. The association of *IL2RA/CD25* with a fifth autoimmune disease suggests that this gene, along with the *PTPN22* gene, appears to play a vital role in immune regulation and function as well as predisposition to autoimmunity in general. Further investigation of the gene using both genetic and functional approaches is now required.

## APPENDIX A: MEMBERS OF THE UKRAG CONSORTIUM, THE BSPAR STUDY GROUP, AND THE YEAR CONSORTIUM

Members of the UKRAG Consortium are as follows: Drs. Anthony G. Wilson, Ann Morgan, Paul Emery, members of the Yorkshire Early Arthritis Register (YEAR) Consortium, Drs. Sophia Steer, Lynne Hocking, David M. Reid, Paul Wordsworth, Pille Harrison, and Deborah Symmons.

Members of the BSPAR Study Group are as follows: Drs. M. Abinum, MD, M. Becker, MD, A. Bell, MD, A. Craft, MD, E. Crawley, MD, J. David, MD, H. Foster, MD, J. Gardener-Medwin, MD, J. Griffin, MD, A. Hall, MD, M. Hall, MD, A. Herrick, MD, P. Hollingworth, MD, L. Holt, MD, S. Jones, MD, G. Pountain, MD, C. Ryder, MD, T. Southwood, MD, I. Stewart, MD, H. Venning. L. Wedderburn, MD, P. Woo, MD, and S. Wyatt, MD.

Members of the YEAR Consortium are as follows: Drs. Paul Emery, Philip Conaghan, Mark Quinn, Ann Morgan, Anne-Maree Keenan, Elizabeth Hensor, Julie Kitcheman (management team); Drs. Andrew Gough, Michael Green, Richard Reece, Lesley Hordon, Philip Helliwell, Richard Melsom, Sheelagh Doherty, Ade Adebajo, Andrew Harvey, Steve Jarrett, Gareth Huson, and Amanda Isdale, Mike Martin, Zinaid Karim, Dennis McGonagle, Colin Pease, and Sally Cox (consultants); Drs. Victoria Bejarano and Jackie Nam (specialist registrars); Claire Brown, Christine Thomas, David Pickles, Alison Hammond, Beverley Neville, Alan Fairclough, Caroline Nunns, Anne Gill, Julie Green, Belinda Rhys-Evans, Barbara Padwell, Julie Madden, Lynda Taylor, Sally Smith, Heather King, Jill Firth, Jayne Heard, Linda Sigsworth (nursing staff); and Diane Corscadden, Karen Henshaw, Lubna-Haroon Rashid, Stephen G. Martin, and James I. Robinson (laboratory staff).
